# Are cervical curvature and axioscapular muscle activity associated with disability in patients with chronic nonspecific neck pain? – a cross sectional exploratory study

**DOI:** 10.3389/fbioe.2024.1441484

**Published:** 2024-10-21

**Authors:** Yanfeng Huang, Aliaa M. Elabd, Roger Adams, Omar M. Elabd, Ahmed A. Torad, Jia Han

**Affiliations:** ^1^ Department of Orthopedics, Jinshan District Central Hospital Affiliated to Shanghai University of Medicine and Health Sciences, Shanghai, China; ^2^ Department of Basic Sciences, Faculty of Physical Therapy, Benha University, Qalubyia, Egypt; ^3^ College of Rehabilitation Sciences, Shanghai University of Medicine and Health Sciences, Shanghai, China; ^4^ Research Institute for Sport and Exercise, University of Canberra, Bruce, ACT, Australia; ^5^ Department of Orthopedics and Its Surgery, Faculty of Physical Therapy, Delta University for Science and Technology, Gamasa, Egypt; ^6^ Department of Physical Therapy, Aqaba University of Technology, Aqaba, Jordan; ^7^ Basic Science Department, Faculty of Physical Therapy, Kafrelsheik University, Kafrelsheik, Egypt; ^8^ Departement of Physical Therapy, Clarkson University, Potsdam, NY, United States

**Keywords:** cross-sectional study, electromyography, neck disability, neck pain, superficial back muscles, spinal curvature

## Abstract

**Purpose:**

To ascertain the relationship between cervical curvature, neck muscle activity and neck disability in patients with chronic nonspecific neck pain (CNNP).

**Methods:**

Ninety participants (mean age = 27.2, female/male ratio = 7/2) with CNNP volunteered. The Neck Disability Index was used to assess neck disability. To indicate the electromyographic characteristics of the axioscapular muscles, the root mean squares and median frequencies of upper trapezius and levator scapula were used. Cervical curvature was measured with a flexible ruler.

**Results:**

Disability of the neck was significantly correlated with curvature (r = −0.599, *p* < 0.001), upper trapezius root mean square (RMS) (r = 0.694, *p* < 0.001) and levator RMS (r = 0.429, *p* < 0.05). Multiple regression analysis produced a significant predictive equation that could predict disability: 33.224− 0.515 × Curvature + 0.156 × Levator RMS − 0.059 × Upper trapezius median frequency + 0.636 × upper trapezius RMS + 0.020 × levator median frequency, with R^2^ = 0.622.

**Conclusion:**

Cervical curvature as well as different axioscapular muscle activity were found to be related to level of disability. These findings have implications for clinical management of CNNP.

## 1 Introduction

One common musculoskeletal condition that affects a large portion of the population is neck pain, which has a major negative impact on people’s quality of life and overall health. According to Cote et al. (2008), prevalence rates are predicted to be between 30% and 50%. Neck pain is a common issue that needs in-depth evaluation and research. While many cases of neck pain are brief and self-limiting, other people suffer from incapacitating, persistent symptoms that significantly affect their everyday activities, posture, and general quality of life (Elabd et al., 2020).

Self-reported disability is a vital component of patient assessment. Measuring the impairment and function associated with neck pain is necessary to evaluate patient outcomes both before and after treatment and to give other stakeholders important information (Howell, 2011). The Neck Disability Index is the most popular form of assessment for neck disability (NDI). Its reliability and validity have been proven (En et al., 2009; Dunleavy et al., 2010; Young et al., 2019). Worldwide, its use is widespread because it has been cross-culturally modified, and the psychometric qualities of its many language translations are similar to those of the original form (Bakhtadze et al., 2015; Lim et al., 2020; Swanenburg et al., 2014).

Understanding all the variables that lead to neck disability and obstruct successful interventions is essential for managing CNNP and related disabilities. Previously, intensity of pain has repeatedly been related to the level of neck disability. However, the recent literature indicates that a variety of physical, psychological, and individual-level factors may influence the likelihood of acquiring neck disability, despite the fact that the exact cause of CNNP and its associated disabilities is not well understood (Kim et al., 2018).

Regarding physical and musculoskeletal factors, a systematic review by Jun et al. (2017) highlighted the need for further research assessing risk variables and their potential to help sufferers avoid or manage neck pain. It has been suggested that one problem is that of neglecting the important role that dysfunction plays and instead concentrating on pathoanatomy as an etiological element of neck pain (Ting et al., 2015). According to Murphy’s theory, pathoanatomy and dysfunction frequently combine to cause clinical symptoms (Murphy, 2000).

Regarding biomechanical dysfunction, the assessment of axioscapular muscle electromyography and cervical sagittal curvature may be important elements. Adopting sustained unbalanced spinal postures with increased neck-shoulder stabilizer activation may be one of the predisposing factors for CNNP (Caneiro et al., 2010). CNNP patients frequently adopt a forward head posture where the cervical spine is moved forward and upward, away from the midline of the body. The bending moment of the head increases pressure applied to the cervical extensors. Further, the cervical extensors become fatigued from continuously maintaining this extended position needed to adjust eye level (Kim and Kim, 2016; Silva et al., 2009).

Previous research has suggested alterations in the electromyographic (EMG) characteristics of the axioscapular muscles in CNNP patients (Zakharova-Luneva et al., 2012). Thus, alterations in axioscapular muscle electromyographic (EMG) characteristics in patients have been considered for the management of CNNP (Elabd et al., 2017). Myofascial trigger points or aberrant cervical spine loading may result from altered axioscapular muscle functioning, which can aggravate neck pain (Sun et al., 2014; Zakharova-Luneva et al., 2012). Further, the repaeting cycle of musculoskeletal dysfunction, or pain-spasm-pain, may be partially explained by the magnitude of the myoelectric signal. Furthermore, a drop in the median frequency of the EMG during task performance may be a sign of muscle fiber exhaustion (Elabd et al., 2017).

Despite the important role of axioscapular muscle electromyography and cervical posture as outcome variables in the management of CNNP, there is little available evidence that highlights their association to the commonly used Neck Disability Index. The need to pinpoint important prognostic factors that can direct clinical judgment and intervention tactics emphasizes the importance of the mission to enhance outcomes for those with persistent, nonspecific neck pain (Weigl et al., 2021). These characteristics allow for the customization of focused treatments that lessen or prevent neck disability in addition to helping to forecast how neck pain will progress.

The goal of this study is to close this research gap by examining the association between Neck Disability Index score, cervical sagittal curvature, and axioscapular muscle electromyography. Both upper trapezius and levator scapula were selected for EMG analysis in this study because they are superficially located, and thus are suitable for EMG study using surface electrodes. For analysis, root mean squares were used to assess muscular activity and median frequencies were used to indicate muscular fatigue. The authors suggest that this study could help to better equip doctors create focused interventions that can lessen or avoid neck impairment in patients with cervical discomfort.

## 2 Materials and methods

### 2.1 Study design

A cross-sectional correlational study was conducted, in compliance with the 1964 Helsinki Affirmation and its subsequent amendments, to investigate the relationships between cervical disability, cervical curvature, and electromyographic (EMG) features (muscle activity and fatigue levels) of the axioscapular muscles (levator scapula and upper trapezius). The Research Ethics Committee of the Faculty of Physical Therapy approved the study at Banha University (PT.BU.EC.7), and it was also filed in the clinical trial registry with the number NCT06301217. Prior to being enrolled in the study, each volunteer provided their written agreement for participation, which was voluntary.

### 2.2 Participants

Individuals with persistent neck pain in the 20–40 years age range were the study’s target population. This range of ages was selected to avoid the anticipated effects of degenerative changes that may occur in the older age. Their diagnosis of chronic neck pain was provided by an orthopaedic surgeon. Respondents to the open invitation completed the first survey. Neck pain has been defined as pain anywhere along the superior nuchal line to the first thoracic spinous process in the posterior part of the cervical spine that had no specific pathology and may or may not radiate to the upper limbs, head, or trunk.

**FIGURE 1:**
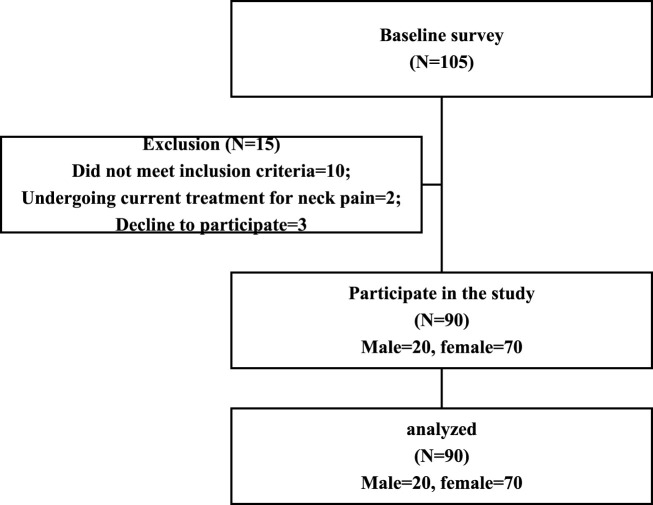
Patient recruitment flowchart.

With over a decade of experience in physiotherapy, the primary author performed standardized physical tests and assessed eligibility requirements. Ninety patients who fulfilled the requirements for eligibility and provided written consent participated in the trial after being specifically told about its risks and procedures. The participant flowchart is displayed in Figure 1.

The eligibility criteria were having mild neck sickness, as indicated by a neck disability index (NDI) score of 15 or higher, and chronic neck discomfort with symptoms that had persisted for at least 3 months (Vernon, 2008). Excluded conditions included cervical spine congenital disorders, disc prolapse, contracture, or surgery; pathologies affecting the cervical spine, such as systemic inflammatory diseases and skin conditions; and visual or auditory problems. Neck pain that was currently being treated medically or through physical therapy was also excluded.

### 2.3 Outcome measures

Prior to the study, anti-inflammatory drugs were to be avoided for 72 h. While the participants completed the self-report questionnaires, an examiner who was not informed of the patient’s allocation collected outcome measures, demographic, and clinical data.

#### 2.3.1 Functional neck disability

Neck disability was measured using the Neck Disability Index (NDI), a 10-item questionnaire. Every participant marked an answer to each question, which ranged from 0 (no disability) to 5 (total disability). After adding up all of the marks and dividing by 50—or 45 if one component was missing—the final score was determined. The validity and reliability of the NDI have already been determined (En et al., 2009; Dunleavy et al., 2010; Swanenburg et al., 2014).

#### 2.3.2 Cervical spine curvature

**FIGURE 2:**
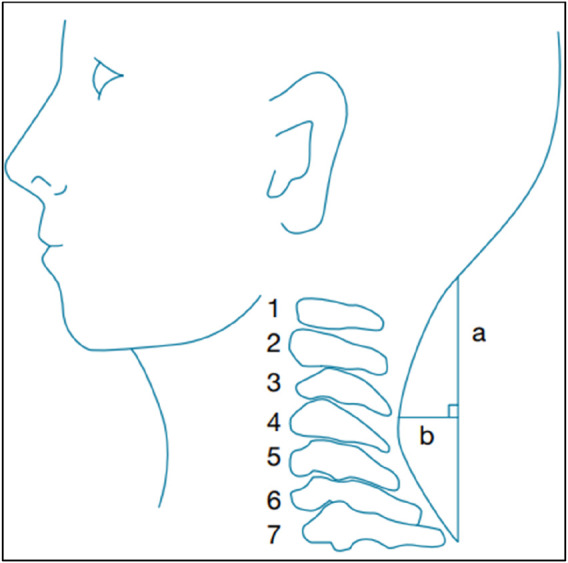
Cervical curve measurement with a flexible ruler. **(A)** The distance measured between the cervical curve’s two endpoints; **(B)** The length of the perpendicular connecting line a’s midpoint to the curve (Elabd et al., 2017).

A flexible ruler (Ati, FC-700R, Taiwan) was employed to determine the cervical spine’s curvature. The ruler is reasonably priced, portable, easy to use, valid, and reliable (Rheault et al., 1989). The patient sat in a chair with his or her feet flat on the floor, with their elbows resting on the armrest directly below the acromion. The patient’s upright cervical spine, between the occiput and the seventh cervical spinous process, was carefully compressed with a flexible ruler to determine the angle. The shape of the flexible ruler (Spinocurve) was traced on a white paper with the endpoints marked. The mathematical equation Angle (Q) = arc tan (2b/a) was applied, as seen in Figure 2 (Elabd et al., 2017).

#### 2.3.3 Electromyographic characteristics

The normalized root mean square (RMS) and median frequency (Elabd et al., 2020) of the upper trapezius and levator scapula were measured using the MyoSystem™ 1400A (Noraxon Inc., 15,770 N. Greenway-Hayden Loop, Suite 100, Scottsdale, United States; E-mail: info@noraxon.com). In order to lower skin impedance, the electrode placement locations had been shaved, and the skin had been cleansed with cotton and alcohol. The patient’s dominant side received two active electrode channels (Elabd et al., 2020). The electrodes were positioned as follows; (A) laterally 2 cm to the midpoint of a line for the upper trapezius between the spinous process of C7 and the posterolateral acromion; B) laterally to the spinous process of C3, 4 between the levator scapula and the upper trapezius; and C) over the C7 spinous process for the reference electrode (McLean, 2005).

Raw EMG signals were amplified and captured with a range of ± 2.5 V. The amplification parameters were as follows; (A) bandwidth = 20–450 Hz; (B) common mode rejection ratio >80 dB at 60 Hz; and (C) input impedance = 10 GΩ. EMG signal systematic bias was eliminated, and they were full-wave corrected before being filtered. Then, Maximum voluntary isometric contractions (MVIC) were used to normalize them. To measure the MVIC, two methods were used: (A) isometric shoulder abduction, where the arm was abducted to 90° and the rotation was neutral for the upper trapezius; and (B) static shoulder elevation, where the shoulder was raised against manual resistance while the levator scapula was maintained in its lateral rotation to the same side. With a 30-s break in between each repetition, every contraction was made three times against manual resistance, held for 7 s each time (McLean, 2005).

After the MVIC evaluation, participants were given the task of writing for 15 min, imposing a semi-static load, since it is the most typical daily work which exacerbates their symptoms. In order to prevent any negative effects on muscle activity during the evaluation, standardised head, neck, shoulder, and spine positions were created by sitting with the back fully supported, feet flat on the floor, and hips and knees flexed to 90° (Szeto et al., 2009). Finally, the raw EMG signals were used to calculate the MDF, with the following calculation for normalized RMS: Writing task EMG amplitude divided by the average of the three MVIC trials yields normalised RMS, which is expressed as a percentage (%) (Nicoletti et al., 2014).

### 2.4 Statistical analysis

Version 25 of SPSS was employed to analyze the data.

After the critical EMG values were identified, a data collection form was used to collect data from each subject. As no missing values were found, no imputation technique was used. After that, to decrease the influence of extreme values, the data were adjusted and examined for outliers. In refining, extreme values are swapped out for values that lie within a specific original distribution percentile. All of the study’s variables were rescaled to convert values that were above the 95th percentile to the 95th percentile value.

The NDI and each variable were correlated using Pearson’s correlation coefficient, and stepwise multiple regression analysis was used to determine the factors influencing NDI. The data were described using the mean and standard deviation.

## 3 Results

Ninety patients (20 males and 70 females) met the eligibility criteria and participated in the study. All of them completed the study and their results were analysed. The length of their pain duration was 5.2 ± 1.5 months and the pain intensity that participants were experiencing was 4.2 ± 0.87 points on the numeric pain rating scale. The descriptives of the sample regarding demographics and NDI were presented in Table 1, while descriptive and correlational statistics for musculoskeletal variables in relation to NDI were presented in Table 2. As a result of the regression analysis, the following equation could be generated:

**TABLE 1:** Descriptives of the sample regarding demographics and NDI.

	Mean	SD
Age	27.2	3.9
Weight	74.3	9.5
Height	1.6	0.1
BMI	28.0	3.3
NDI	23.0	9.0

SD, standard deviation.

**TABLE 2:** Descriptive statistics and correlational statistics showing relationship to NDI.

	Mean	SD	Correl (CI)	Sig
Curvature	28.04	7.04	−0.599 (−0.717–−0.447)	<0.001
LV_NOR_RMS	12.13	10.16	0.429 (0.244–0.585)	0.025
UT_MDF	68.65	18.88	−0.489 (−0.632–−0.314)	0.17
UT_NOR_RMS	7.92	5.79	0.694 (0.568–0.788)	<0.001
LV_MDF	65.44	15.58	−0.441 (−0.594–−0.257)	0.695

CI, confidence interval; Correl, correlation with NDI; SD, standard deviation; MDF, median frequency; RMS, root mean square.

NDI = 33.224−0.515 × Curve + 0.156 × LV_NOR_RMS−0.059 × UT_MDF + 0.636 × UT_NOR_RMS + 0.020 × LV_MDF.

This model had an Adjusted R Square 0.622.

## 4 Discussion

Our results suggest that upper trapezius root mean square is a strong predictor of neck disability (Caneiro et al., 2010). This may be explained as high root mean square (RMS) values in electromyography (EMG) studies of the upper trapezius muscle indicate elevated muscle activation, which can lead to muscle strain and fatigue. This may contribute to neck pain and disability by impairing muscle function and increasing the risk of musculoskeletal disorders (Patselas et al., 2021). Also, the upper trapezius is critical for maintaining neck and shoulder posture. Excessive activation, as indicated by high RMS, may reflect poor ergonomic practices or prolonged postural stress, leading to neck pain and disability (Torad et al., 2024; Wolff et al., 2022). In addition, elevated trapezius muscle activity can lead to nerve entrapment and reduced blood flow within the muscle, causing pain and contributing to disability through mechanisms of ischemia and sensitization of pain receptors (Urits et al., 2020).

Our results suggest that cervical curvature is strongly associated with neck disability (Caneiro et al., 2010). This could be explained because an abnormal cervical curve (e.g., hyperlordosis) can increase biomechanical stress on the cervical spine, leading to degenerative changes, pain, and disability by altering the distribution of forces across the cervical vertebrae (Le Huec et al., 2019). Further, changes in the cervical curve can lead to neural compression, affecting nerve root function and contributing to symptoms of pain, numbness, and weakness, which are markers of neck disability (Winger, 2022). Another explanation is that an altered cervical curvature can result in muscle tension and imbalance, with certain muscles becoming overactivated to compensate for the abnormal curvature, leading to fatigue, pain, and functional limitations (Treff, 2014).

Our results suggest that levator scapulae root mean square (RMS) is less strongly associated with neck disability (0.025) compared to the upper trapezius RMS, which can be readily physiologically justified. The upper trapezius muscle plays a more direct role in supporting the neck and head due to its larger size and strategic location. It is often more involved in postural support and stabilization, making its activity levels more closely related to neck strain and discomfort. In contrast, the levator scapulae, while important for neck and shoulder movement, has a more specialized role that may not directly correlate with general neck disability to the same extent (Baghi et al., 2017).

The biomechanical stress across the cervical region may be more significantly influenced by the upper trapezius due to its extensive attachments and role in load distribution. High RMS values in the upper trapezius could indicate excessive stress and potential for injury, which directly impacts neck health. The levator scapulae, having a more localized function, might not exhibit the same level of predictive value for overall neck disability (Hemant et al., 2013).

Variations in posture and ergonomic factors may have a more pronounced impact on the upper trapezius muscle due to its involvement in a wide range of movements and postural maintenance. The levator scapulae’s contribution to neck disability might be more specific to certain movements or positions, making its RMS a less strong overall predictor of neck disability (Varol et al., 2024). Upper trapezius and levator median frequencies are poorly associated with neck disability (0.17, 0.695) and this result may be due to the median frequency in EMG signals often being used to assess muscle fatigue. However, poor correlation with neck disability may indicate that these frequencies do not capture the specific aspects of muscle function or dysfunction that may lead to disability (Phinyomark et al., 2012). Another prospective account is that there is significant individual variability in median frequencies due to factors like muscle fiber composition and physical condition, making it a relatively poor universal predictor of neck disability (Baguet et al., 2011). Finally, individuals may develop compensatory mechanisms that alter median frequency values in a way that does not directly correlate with disability levels, such as by changing their posture or utilizing other muscle groups to reduce strain on the neck (Meisingset et al., 2016).

Based on the results of the current study, workplace and lifestyle modifications should be prioritized to reduce prolonged muscle strain, especially in the upper trapezius. Ergonomic assessments can help identify risk factors contributing to increased muscle activity and subsequent neck disability. Furthermore, rehabilitation programs should include exercises aimed at reducing muscle tension and improving posture. Strengthening and stretching exercises for the upper trapezius and levator scapulae, along with exercises promoting overall cervical spine health, could be beneficial.

Future research should focus on longitudinal studies, so as to better understand the causal relationships between muscle activity and neck disability. This approach may help in identifying early predictors of neck pain and disability, facilitating preventative strategies. In addition, there is a need for randomized controlled trials evaluating the effectiveness of specific interventions targeting muscle activity, such as biofeedback, ergonomic adjustments, and therapeutic exercises, in reducing neck disability.

### 4.1 Limitations

Despite its strengths, the study has some limitations that should be taken into account; (A) the small sample size could make it harder to extrapolate our results to a larger population; (B) the study focused specifically on a particular population of patients with cervical pain, and it remains uncertain whether these models would yield similar performance in other populations with different pain types or disabilities; and (C) the study examined only a limited set of contributors and there may be additional factors not included in our analysis that could also be important in predicting disability among these patients.

### 4.2 Conclusion

To variable degrees, cervical curvature and the activity of the axioscapular muscles were associated to neck disability. However, the median frequencies of these muscles appear to be poor predictors of neck disability, indicating the complexity of factors that contribute to neck health. This study highlights the multifaceted nature of neck disability and the need for comprehensive assessment strategies in clinical practice.

## Data Availability

The raw data supporting the conclusions of this article will be made available by the authors, without undue reservation.

## References

[B1] Baghi R. Rahnama L. Karimi N. Goodarzi F. Rezasoltani A. Jaberzadeh S. (2017). Differential activation of the dorsal neck muscles during a light arm-elevation task in patients with chronic nonspecific neck pain and asymptomatic controls: an ultrasonographic study. PM R. 9, 699–706. 10.1016/j.pmrj.2016.10.020 27836771

[B2] Baguet A. Everaert I. Hespel P. Petrovic M. Achten E. Derave W. (2011). A new method for non-invasive estimation of human muscle fiber type composition. PLoS One 6, e21956. 10.1371/journal.pone.0021956 21760934 PMC3131401

[B3] Bakhtadze M. A. Vernon H. Zakharova O. B. Kuzminov K. O. Bolotov D. A. (2015). The neck disability index-Russian language version (NDI-RU): a study of validity and reliability. Spine (Phila Pa 1976) 40, 1115–1121. 10.1097/BRS.0000000000000880 25768684

[B4] Caneiro J. P. O’Sullivan P. Burnett A. Barach A. O’Neil D. Tveit O. (2010). The influence of different sitting postures on head/neck posture and muscle activity. Man. Ther. 15, 54–60. 10.1016/j.math.2009.06.002 19643658

[B5] Cote P. van der Velde G. Cassidy J. D. Carroll L. J. Hogg-Johnson S. Holm L. W. (2008). The burden and determinants of neck pain in workers: results of the bone and joint decade 2000-2010 task force on neck pain and its associated disorders. Spine (Phila Pa 1976) 33, S60–S74. 10.1097/BRS.0b013e3181643ee4 18204402

[B6] Dunleavy K. Mariano H. Wiater T. Goldberg A. (2010). Reliability and minimal detectable change of spinal length and width measurements using the Flexicurve for usual standing posture in healthy young adults. J. Back Musculoskelet. Rehabil. 23, 209–214. 10.3233/BMR-2010-0269 21079300

[B7] Elabd A. Ibrahim A. Elhafez H. (2017). Kinesio taping versus postural correction exercises on mechanically triggered neck dysfunction. Int. J. Ther. Rehabilitation 24, 155–162. 10.12968/ijtr.2017.24.4.155

[B8] Elabd A. M. Ibrahim A. R. Elhafez H. M. Hussien H. A. Elabd O. M. (2020). Efficacy of kinesio taping and postural correction exercises on levator scapula electromyographic activities in mechanical cervical dysfunction: a randomized blinded clinical trial. J. Manip. Physiol. Ther. 43, 588–596. 10.1016/j.jmpt.2019.05.010 32709516

[B9] En M. C. Clair D. A. Edmondston S. J. (2009). Validity of the neck disability index and neck pain and disability scale for measuring disability associated with chronic, non-traumatic neck pain. Man. Ther. 14, 433–438. 10.1016/j.math.2008.07.005 18824393

[B10] Hemant P. N. Suhas M. B. Anil M. A. (2013). Restoration of normal length of upper trapezius and levator scapulae in subjects with adhesive capsulitis. Indian J. Physiother. Occup. Ther. 7, 141.

[B11] Howell E. R. (2011). The association between neck pain, the Neck Disability Index and cervical ranges of motion: a narrative review. J. Can. Chiropr. Assoc. 55, 211–221. 21886283 PMC3154067

[B12] Jun D. Zoe M. Johnston V. O’Leary S. (2017). Physical risk factors for developing non-specific neck pain in office workers: a systematic review and meta-analysis. Int. Arch. Occup. Environ. Health 90, 373–410. 10.1007/s00420-017-1205-3 28224291

[B13] Kim E.-K. Kim J. S. (2016). Correlation between rounded shoulder posture, neck disability indices, and degree of forward head posture. J. Phys. Ther. Sci. 28, 2929–2932. 10.1589/jpts.28.2929 27821964 PMC5088155

[B14] Kim R. Wiest C. Clark K. Cook C. Horn M. (2018). Identifying risk factors for first-episode neck pain: a systematic review. Musculoskelet. Sci. Pract. 33, 77–83. 10.1016/j.msksp.2017.11.007 29197234

[B15] Le Huec J.-C. Thompson W. Mohsinaly Y. Barrey C. Faundez A. (2019). Sagittal balance of the spine. Eur. Spine J. 28, 1889–1905. 10.1007/s00586-019-06083-1 31332569

[B16] Lim H. H. R. Tang Z. Y. Hashim M. Yang M. Koh E. Y. L. Koh K. H. (2020). Cross-cultural adaptation, reliability, validity, and responsiveness of the simplified-Chinese version of neck disability index. Spine (Phila Pa 1976) 45, 541–548. 10.1097/BRS.0000000000003325 31770333 PMC7208282

[B17] McLean L. (2005). The effect of postural correction on muscle activation amplitudes recorded from the cervicobrachial region. J. Electromyogr. Kinesiol 15, 527–535. 10.1016/j.jelekin.2005.06.003 16150608

[B18] Meisingset I. Stensdotter A. K. Woodhouse A. Vasseljen O. (2016). Neck motion, motor control, pain and disability: a longitudinal study of associations in neck pain patients in physiotherapy treatment. Man. Ther. 22, 94–100. 10.1016/j.math.2015.10.013 26586133

[B19] Murphy D. R. (2000). Conservative management of cervical spine syndromes. New York, NY: McGraw-Hill.

[B20] Nicoletti C. Spengler C. M. Laubli T. (2014). Physical workload, trapezius muscle activity, and neck pain in nurses’ night and day shifts: a physiological evaluation. Appl. Ergon. 45, 741–746. 10.1016/j.apergo.2013.09.016 24140243

[B21] Patselas T. Karanasios S. Sakellari V. Fysekis I. Patselas M. I. Gioftsos G. (2021). EMG activity of the serratus anterior and trapezius muscles during elevation and PUSH UP exercises. J. Bodyw. Mov. Ther. 27, 247–255. 10.1016/j.jbmt.2021.02.002 34391241

[B22] Phinyomark A. Thongpanja S. Hu H. Phukpattaranont P. Limsakul C. (2012). “The usefulness of mean and median frequencies in electromyography analysis,” in Computational intelligence in electromyography analysis-A perspective on current applications and future challenges. Editor Naik G. R. (InTech), 195–220.

[B23] Rheault W. Ferris S. Foley J. A. Schaffhauser D. Smith R. (1989). Intertester reliability of the flexible ruler for the cervical spine. J. Orthop. Sports Phys. Ther. 10, 254–256. 10.2519/jospt.1989.10.7.254 18791322

[B24] Silva A. G. Punt T. D. Sharples P. Vilas-Boas J. P. Johnson M. I. (2009). Head posture and neck pain of chronic nontraumatic origin: a comparison between patients and pain-free persons. Archives Phys. Med. rehabilitation 90, 669–674. 10.1016/j.apmr.2008.10.018 19345785

[B25] Sun A. Yeo H. G. Kim T. U. Hyun J. K. Kim J. Y. (2014). Radiologic assessment of forward head posture and its relation to myofascial pain syndrome. Ann. Rehabil. Med. 38, 821–826. 10.5535/arm.2014.38.6.821 25566482 PMC4280379

[B26] Swanenburg J. Humphreys K. Langenfeld A. Brunner F. Wirth B. (2014). Validity and reliability of a German version of the neck disability index (NDI-G). Man. Ther. 19, 52–58. 10.1016/j.math.2013.07.004 23920153

[B27] Szeto G. P. Straker L. M. O’Sullivan P. B. (2009). Examining the low, high and range measures of muscle activity amplitudes in symptomatic and asymptomatic computer users performing typing and mousing tasks. Eur. J. Appl. Physiol. 106, 243–251. 10.1007/s00421-009-1019-4 19255772

[B28] Ting L. H. Chiel H. J. Trumbower R. D. Allen J. L. McKay J. L. Hackney M. E. (2015). Neuromechanical principles underlying movement modularity and their implications for rehabilitation. Neuron 86, 38–54. 10.1016/j.neuron.2015.02.042 25856485 PMC4392340

[B29] Torad A. A. Ahmed M. M. Elabd O. M. El-Shamy F. F. Alajam R. A. Amin W. M. (2024). Identifying predictors of neck disability in patients with cervical pain using machine learning algorithms: a cross-sectional correlational study. J. Clin. Med. 13, 1967. 10.3390/jcm13071967 38610732 PMC11012682

[B30] Treff M. (2014). An investigation of musculoskeletal imbalances in the thoracic and cervical regions, with respect to an improved diagnostic approach for upper crossed syndrome. Master Thesis.

[B31] Urits I. Charipova K. Gress K. Schaaf A. L. Gupta S. Kiernan H. C. (2020). Treatment and management of myofascial pain syndrome. Best. Pract. Res. Clin. Anaesthesiol. 34, 427–448. 10.1016/j.bpa.2020.08.003 33004157

[B32] Varol U. Valera-Calero J. A. Sánchez-Jiménez E. Fernández-de-Las-Peñas C. Ortega-Santiago R. Kobylarz M. D. (2024). Levator scapulae stiffness measurement reliability in individuals with and without chronic neck pain by experienced and novel examiners. Sensors 24, 277. 10.3390/s24010277 38203140 PMC10781297

[B33] Vernon H. (2008). The neck disability index: state-of-the-art, 1991-2008. J. Manip. Physiol. Ther. 31, 491–502. 10.1016/j.jmpt.2008.08.006 18803999

[B34] Weigl M. Letzel J. Angst F. (2021). Prognostic factors for the improvement of pain and disability following multidisciplinary rehabilitation in patients with chronic neck pain. BMC Musculoskelet. Disord. 22, 330. 10.1186/s12891-021-04194-9 33812386 PMC8019506

[B35] Winger J. (2022). Disorders of the neck and back. Family medicine: principles and practice. New York: Springer, 1551–1567.

[B36] Wolff W. Heinemann C. M. Lipps D. B. (2022). The influence of idiopathic chronic neck pain on upper trapezius and sternocleidomastoid muscle activity and elasticity during functional reaching: a cross-sectional study. J. Biomech. 141, 111223. 10.1016/j.jbiomech.2022.111223 35926366

[B37] Young I. A. Dunning J. Butts R. Mourad F. Cleland J. A. (2019). Reliability, construct validity, and responsiveness of the neck disability index and numeric pain rating scale in patients with mechanical neck pain without upper extremity symptoms. Physiother. Theory Pract. 35, 1328–1335. 10.1080/09593985.2018.1471763 29856244

[B38] Zakharova-Luneva E. Jull G. Johnston V. O’Leary S. (2012). Altered trapezius muscle behavior in individuals with neck pain and clinical signs of scapular dysfunction. J. Manip. Physiol. Ther. 35, 346–353. 10.1016/j.jmpt.2012.04.011 22608287

